# RAN translation as a dominant pathogenic axis in C9ORF72-associated ALS and FTD models

**DOI:** 10.1186/s13024-026-00959-9

**Published:** 2026-06-06

**Authors:** Pegah Masrori, Defne Audrey Amado

**Affiliations:** 1 Department of Neurology, Cliniques Universitaires Saint-Luc, Brussels, Belgium; 2 Institute of Neuroscience, UCLouvain, Brussels, Belgium; 3 Department of Neurology, Perelman School of Medicine, University of Pennsylvania, Philadelphia, PA USA; 4 Raymond G. Perelman Center for Cellular and Molecular Therapeutics, Children’s Hospital of Philadelphia, Philadelphia, PA USA

Since the identification of the GGGGCC hexanucleotide repeat expansion in *C9ORF72* as the most common genetic cause of amyotrophic lateral sclerosis (ALS) and frontotemporal dementia (FTD) in European and North American populations [1, 2], a central mechanistic question has dominated the field: does neurodegeneration arise primarily from toxicity of the expanded repeat RNA, or from the dipeptide repeat proteins (DPRs) generated by repeat-associated non-AUG (RAN) translation [3]?.

Despite more than a decade of intensive investigation, disentangling these mechanisms has proven elusive, leaving pathogenic causality unresolved and therapeutic strategies diffuse.

In this issue of *Science*, Jiang and colleagues [4] make a significant stride in resolving this debate by genetically uncoupling repeat RNA accumulation from RAN translation. By disrupting a single CUG codon that functions as a dominant initiation site for RAN translation, the authors markedly reduce DPR production while preserving repeat RNA expression and nuclear RNA foci. The resulting rescue of molecular, cellular, and behavioral phenotypes across multiple *C9ORF72* mouse models and the partial rescue in patient-derived neurons demonstrates that DPRs are not ancillary contributors but are major drivers of the measured phenotypes in these models of neurodegeneration. Mechanistically, several DPR species, particularly poly(GR) and poly(PR), have been shown to disrupt nucleocytoplasmic transport, impair ribosomal function, and alter stress granule dynamics. Arginine-rich DPRs have additionally been shown to perturb liquid–liquid phase separation and nucleolar function, providing plausible links between RAN translation and downstream neurodegenerative processes.

The prevailing “dual-toxicity” model has proposed that *C9ORF72*-associated disease emerges from the combined effects of RNA-mediated and protein-mediated toxicity [3, 5–7]. While conceptually appealing, this framework has been experimentally limiting: most genetic or pharmacological interventions reduce both repeat RNA and DPRs simultaneously, precluding definitive causal inference. Jiang et al. [4] overcome this barrier through a precise genetic strategy that markedly reduces translation from repeat RNAs without altering their abundance or nuclear localization, and which furthermore preserves the structure of the expanded repeat with the exception of localized structure changes near the mutation site. The persistence of abundant RNA foci in phenotypically rescued systems compels a reassessment of their pathogenic role. Although expanded repeat RNAs have been shown to sequester RNA-binding proteins and perturb nuclear homeostasis [5, 8, 9], these findings suggest that such effects may be insufficient to drive neurodegeneration below a threshold of DPR production. RNA foci may therefore represent a molecular hallmark of the repeat expansion rather than its proximate toxic effector. An important caveat to this conclusion is that the relative contribution of RNA-mediated toxicity in human disease may depend on cellular context and disease stage, and cannot therefore be fully excluded based on the current data.

RAN translation has been documented across multiple repeat expansion disorders [10], yet its status as a primary pathogenic mechanism has remained uncertain. This study [4] strengthens the case that RAN translation is a major driver of disease-relevant phenotypes across multiple *C9ORF72*-associated ALS and FTD models. The identification of a single non-AUG initiation codon that dominates DPR synthesis across reading frames, at least for the sense strand, argues against a stochastic model of aberrant translation, instead suggesting that a substantial component of RAN translation may depend on defined initiation events governed by discrete molecular checkpoints. These findings support a model in which translational output accounts for a substantial fraction of the measurable toxicity associated with repeat-containing RNAs [6]. In this model, aberrant translation, rather than transcription, may therefore be the critical inflection point linking repeat expansion to neurodegeneration. Importantly, antisense-derived transcripts and DPR species were not directly targeted in this approach and may contribute independently to disease mechanisms.

The therapeutic implications are immediate and consequential. RNA-targeting strategies, including antisense oligonucleotides, have shown variable efficacy in preclinical and clinical settings [11–13], with multiple possible causes including deleterious effects of loss of endogenous C9orf72 function, incomplete suppression of DPR synthesis despite reductions in repeat RNA levels, off-target effects of the therapeutic, or issues with timing or target engagement. In addition, effective therapeutic strategies may require coordinated targeting of both sense and antisense transcripts to achieve sufficient suppression of toxic species. The findings of Jiang et al. raise the possibility that therapeutic efficacy may depend, at least in part, on the extent to which RAN translation is suppressed [4]. Conversely, selectively targeting RAN translation offers a rational route to neutralize toxicity while preserving endogenous *C9ORF72* expression, an important consideration given the gene’s roles in immune regulation and lysosomal function [14, 15]. These observations motivate further evaluation of approaches aimed at modulating RAN translation, including initiation-site editing, translation-factor targeting, and small-molecule strategies.

Although this work indicates that DPR production drives several phenotypes in the studied models, it sharpens several critical questions. How do DPRs contribute to TDP-43 pathology, neuroinflammation, and neuronal loss? Does RAN translation operate upstream of TDP-43 mislocalization and aggregation, or does it represent a parallel pathogenic axis? Are specific DPR species disproportionately pathogenic, or does suppression of a common initiation event collapse an interconnected toxic network? Crucially, whether inhibition of RAN translation can halt or reverse established pathology remains unknown and will determine its ultimate translational relevance.

Beyond *C9ORF72*, these findings prompt a broader reassessment of repeat expansion disorders. In *C9ORF72*, rather than viewing RNA and protein toxicity as parallel or additive processes, this work supports a hierarchical model in which aberrant translation may represent the dominant pathogenic mechanism. The extent to which this model applies across the broader group of repeat expansion disorders, which affect vastly different brain and body regions and cell types, remains to be elucidated. In myotonic dystrophy type 1 (DM1) and type 2 (DM2), repeat expansion disorders primarily affecting muscle, pathogenesis stems from the repeat expansion-containing RNA sequestering MBNL, causing broad downstream mis-splicing [16]. Do other repeat expansion disorders behave more like *C9orf72*, in which translation is the effector, or like DM1, in which RNA-mediated effects predominate? Does the behavior of each disorder depend on the length of the repeat, the cell type affected, structural specifics of the nucleotide sequence, or other factors? Perhaps of equal importance to the specific findings presented here is the fact that the sequence of experiments conducted by Jiang and colleagues can be applied to address these questions. This in turn can determine the extent to which RAN translation acts as a unifying and therapeutically actionable principle across genetically diverse neurodegenerative diseases.

In sum, Jiang et al. [4] provide compelling evidence that suppressing RAN translation can rescue a broad range of molecular, cellular, and behavioral phenotypes across multiple *C9ORF72* disease models while preserving repeat RNA expression and RNA foci. These findings substantially strengthen the case that DPR production is a major driver of toxicity in the studied systems. Although the roles of antisense transcripts, RNA-mediated toxicity, C9ORF72 haploinsufficiency, and the relationship between DPR suppression and TDP-43 pathology in human disease remain to be elucidated, the findings here argue for addressing RAN translation in future therapeutic strategies. As the field advances toward precision interventions for repeat expansion disorders, targeting aberrant translation in *C9ORF72-*ALS/FTD may represent the most direct route from molecular insight to clinical impact.

## Data Availability

Not applicable.
